# Structure calculation, refinement and validation using *CcpNmr Analysis*

**DOI:** 10.1107/S1399004714026662

**Published:** 2015-01-01

**Authors:** Simon P. Skinner, Benjamin T. Goult, Rasmus H. Fogh, Wayne Boucher, Tim J. Stevens, Ernest D. Laue, Geerten W. Vuister

**Affiliations:** a Department of Biochemistry, University of Leicester, Lancaster Road, Leicester LE1 9HN, England; b Department of Biochemistry, University of Cambridge, Tennis Court Road, Cambridge CB2 1GA, England; c Cell Biology Division, MRC Laboratory of Molecular Biology, Francis Crick Avenue, Cambridge CB2 0QH, England

**Keywords:** NMR, processing, structure calculation, analysis, *CcpNmr*, talin

## Abstract

*CcpNmr Analysis* provides a streamlined pipeline for both NMR chemical shift assignment and structure determination of biological macromolecules. In addition, it encompasses tools to analyse the many additional experiments that make NMR such a pivotal technique for research into complex biological questions. This report describes how *CcpNmr Analysis* can seamlessly link together all of the tasks in the NMR structure-determination process. It details each of the stages from generating NMR restraints [distance, dihedral, hydrogen bonds and residual dipolar couplings (RDCs)], exporting these to and subsequently re-importing them from structure-calculation software (such as the programs *CYANA* or *ARIA*) and analysing and validating the results obtained from the structure calculation to, ultimately, the streamlined deposition of the completed assignments and the refined ensemble of structures into the PDBe repository. Until recently, such solution-structure determination by NMR has been quite a laborious task, requiring multiple stages and programs. However, with the new enhancements to *CcpNmr Analysis* described here, this process is now much more intuitive and efficient and less error-prone.

## Abstract 2

This report describes the working of the program *CcpNmr Analysis* for both NMR chemical shift assignment and structure determination of biological macromolecules.

## 1. Introduction   

**Figure 1:**
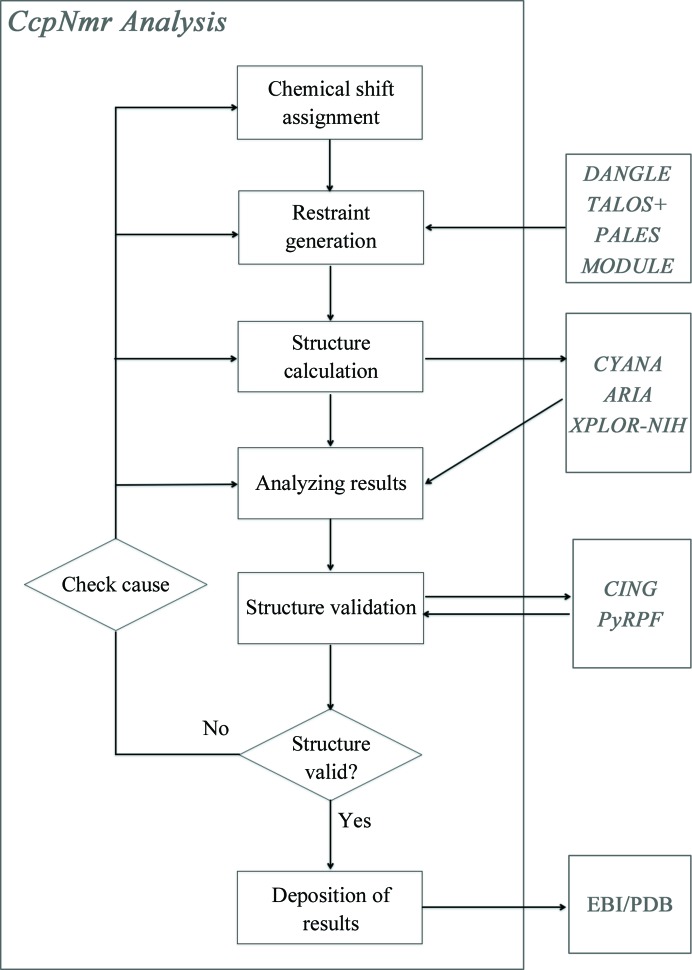
Flowchart of the NMR structure-determination pipeline that *CcpNmr Analysis* facilitates. See the text for a discussion of the different aspects of the process. Programs or web-based services external to the *CcpNmr Analysis* program (grey boxes) facilitate specific tasks such as structure calculations or structure validation.

This report is formed of two parts; first we will describe the theory and application of each of the stages of the NMR structure-determination process (for a review, see Vranken *et al.*, 2015 ▶) using the *CcpNmr Analysis* software package (Vranken *et al.*, 2005 ▶; Fig. 1 ▶). Subsequently, to illustrate the *CcpNmr Analysis* workflow we describe a case study in which the program was central to the analysis of our data and has impacted on the research in our laboratory, namely the protein talin.

Typically, an NMR structure determination involves multiple cycles of data analysis, structure calculation and structural assessment. Over the course of the development of the *CcpNmr Analysis* program, we concluded that the preferred practices of individual researchers vary considerably. Consequently, we designed the structure-calculation framework of *CcpNmr Analysis* to be flexible in its interface for setting up calculations, as well as to be adaptable in the choice of protocols used. This includes a flexible choice of which resource to use for the calculations, the need to customize the data elements used in the calculations and the specification of the data to be imported back into *CcpNmr Analysis* for further inspection. The scheme currently implemented in *CcpNmr Analysis* allows dedicated, flexible tools that aid the researcher in progressing through each of the steps (detailed below). The program is suitable for the analysis of NMR data from both proteins and oligonucleotides, although some of the tools can be specific to either category.

## 2. Workflow using *CcpNmr Analysis*   

### 2.1. Restraint generation   

A typical NMR structure calculation involves the generation of restraints that are used as input for structure-generation programs. There are four main types of NMR restraint: distances, dihedral angles, hydrogen bonds and orientational restraints such as RDCs (for a review, see Vuister *et al.*, 2011 ▶). *CcpNmr Analysis* is able to generate and handle all of these.

#### 2.1.1. Distance restraints   

Generation of distance restraints using *CcpNmr Analysis* is extremely easy. The process is based upon the *r* ^−6^ distance dependence of the NOE (Abragam, 1961 ▶) and involves using peaks corresponding to known distances for calibration. More intense resonances from methyl groups are treated as three overlapping single-proton peaks. The calibration parameters must be set by the user, *e.g.* using peaks corresponding to known distances in secondary structures. As a rough heuristic and initial approximation, *CcpNmr Analysis* derives a default setting such that the average peak intensity corresponds to a distance of 3.2 Å. Actual distance bounds are calculated as a fraction of the target value, either using the peak intensities or directly from the derived distance. It must be noted that common *r* ^−6^ averaging protocols for the NOE-derived distance restraints nowadays tend to use 0 Å lower bounds. The calibration procedure relies on various assumptions that are not always completely fulfilled, *e.g.* that the NOE build-up rate is linear at the chosen NOE mixing time, as well as the absence of non-isotropic or local dynamical processes. Nevertheless, in practice the *r* ^−6^ distance dependence of the calculation makes the distance derivation very robust in the face of such calibration approximations.

Assigned distance restraints can be calculated from solution-state NOESY and solid-state spectra using the ‘Make Distance Restraints’ command that queries for the relevant parameters (including the relative width of the allowed distance range) using a dedicated ‘popup’ window. This generation is performed for one input spectrum at a time, after which the resulting restraint lists can be merged into a single list, which can be subsequently separated into non-ambiguous and ambiguous restraints (*i.e.* restraints involving multiple possibilities) with a single click. Further, the ‘Shift Match Restraints’ facility matches chemical shifts of resonances to unassigned peak positions and thus can produce highly ambiguous distance restraints from the peaks to be assessed by the subsequent structure-calculation algorithm. Distance restraints can also be calculated with correction for specific isotope-labelling schemes (Atreya, 2012 ▶), which is especially useful when handling solid-state NMR data (Stevens *et al.*, 2011 ▶).

*CcpNmr Analysis* also contains a facility for calibration of distance restraints with respect to a reference spectrum *via* the peak-normalization section of the ‘Make Distance Restraints’ popup. This facility automatically scales distance restraints derived from the NOE spectrum using the relative intensity information derived from another spectrum. For example, if specific residues display varying peak intensities in an HSQC spectrum, the corresponding peaks in the NOESY-HSQC spectrum would be scaled to the same degree using this facility.

Distance restraints may optionally be improved using the so-called network-anchoring function (Herrmann *et al.*, 2002 ▶), which operates over several peak lists at one time to produce a single set of vetted distance restraints. The essence of this approach is that the correctly assigned restraints form a self-consistent subset of the network of restraints.

Using a set of derived distance restraints, an ensemble of structure models can be generated in a semi-automated manner. Alternatively, structure calculations can be carried out by directly using peak lists from NMR spectra, for example using the *CYANA*/*CANDID* (Güntert *et al.*, 1997 ▶; Herrmann *et al.*, 2002 ▶; Güntert, 2009 ▶) or *ARIA* (Rieping *et al.*, 2007 ▶) software packages.

#### 2.1.2. Chemical shift-derived dihedral angles   

Chemical shifts are a valuable source of structural information (Spera & Bax, 1991 ▶). Predictions of dihedral angles on the basis of chemical shifts can be obtained using the *TALOS*+ program (Shen *et al.*, 2009 ▶) or the *DANGLE* (*Dihedral ANgles from Global Likelihood Estimate*) algorithm (Cheung *et al.*, 2010 ▶). The latter is fully integrated into *CcpNmr Analysis* and thus allows dihedral angle prediction with a single button click. Once dihedral angles have been predicted by *DANGLE*, the resulting per-residue likelihood estimates are displayed as Ramachandran plots. This facility enables the user to analyse and select reliable predictions prior to committing these into a dihedral restraint list within the *CcpNmr Analysis* project.

#### 2.1.3. Hydrogen bonds   

Hydrogen bonds can be detected using different NMR techniques, including H/D exchange (Englander & Kallenbach, 2009 ▶), measurement of long-range ^3^ *J* couplings (Blackledge, 2007 ▶) and the measurement of an HSQC temperature series (Baxter *et al.*, 1998 ▶). While *CcpNmr Analysis* does not have a dedicated hydrogen-bond determination module, once these data have been collected and analysed elsewhere *CcpNmr Analysis* does provide a tool to simplify entering a set of hydrogen-bond restraints by selecting the appropriate atoms and distance limits.

#### 2.1.4. Orientational restraints   

Orientational restraints such as RDCs contain valuable information as they report on the orientation of bond vectors relative to an overall molecular frame (Lipsitz & Tjandra, 2004 ▶). Their use in structure-calculation protocols is becoming more and more common, and *CcpNmr Analysis* implements several methods for analysing the underlying NMR data as well as providing routines for calculation of the restraints. Macros have been developed for the analysis of in-phase/antiphase (IPAP) NMR data (Ottiger *et al.*, 1998 ▶), and the programs *PALES* (Zweckstetter, 2008 ▶) and *MODULE* (Dosset *et al.*, 2001 ▶) are integrated into *CcpNmr Analysis*.

### 2.2. Structure calculation   

*CcpNmr Analysis* is designed for NMR data analysis and functions as an interface to external structure-calculation programs. Hence, all structure calculations, either *in vacuo* or in explicit water, are performed using the specific protocols implemented by these external programs. To function as an interface, all restraint lists generated in *CcpNmr Analysis* can be exported in a variety of different formats, including those suitable for the common structure-calculation programs *ARIA* (Rieping *et al.*, 2007 ▶), *CYANA* (Güntert *et al.*, 1997 ▶; Herrmann *et al.*, 2002 ▶; Güntert, 2009 ▶) and *XPLOR-NIH* (Schwieters *et al.*, 2003 ▶), using *CcpNmr Format Converter* (Vranken *et al.*, 2005 ▶) and then used as input for the calculation. Alternatively, structure calculations can be initiated from within *CcpNmr Analysis* in a highly streamlined manner.

Interfaces for both the *CYANA* (Güntert *et al.*, 1997 ▶; Herrmann *et al.*, 2002 ▶; Güntert, 2009 ▶) and the *ARIA* (which natively includes water refinement; Rieping *et al.*, 2007 ▶) structure-calculation programs have been incorporated into *CcpNmr Analysis*, enabling these calculations to be set up and executed with considerable ease. To execute a structure calculation from within *CcpNmr Analysis*, the user needs only to select the input data and, in the case of *CYANA*, some rudimentary parameters such as the number of output structures and the residue ranges for r.m.s.d. calculations. The user can easily customize the parameters that are to be queried in the setup of the calculation, as all such definitions are contained within a simple, user-adaptable protocol-definition file.

Once parameters have been set and the NMR restraint lists to be used in the calculation have been selected by the user, the latter are automatically exported in the correct format using *CcpNmr Format Converter* (Vranken *et al.*, 2005 ▶). The user-selected parameter values are used to generate the scripts required to perform the calculation and *CcpNmr Analysis* also generates the required information for the re-import of the results once the calculations are completed. This information is stored in a small calculation-definition file within the directory that contains the input and calculation results data.

After preparation of all of the data, the calculation can be executed on the user’s host machine or alternatively on any other computational resource, such as a local cluster or an external grid (*e.g.* WeNMR; Wassenaar *et al.*, 2012 ▶). The result of an NMR structure calculation is an ensemble of typically ∼20 structural conformers consistent with the input data, plus additional data detailing specific aspects of the computation such as the links between the experimental data and automatically derived restraints.

On completion of a structure calculation, all of the output data are seamlessly and faithfully imported back into *CcpNmr Analysis* automatically, provided that the above-mentioned calculation-definition file is still part of the data and import is initiated from the original *CcpNmr Analysis* project or a direct copy. Importantly, in the case of *CYANA* calculations its calculation-overview file (final.ovw) and the data contained in the ‘cyanatable’ file are also imported into *CcpNmr Analysis* to allow easy inspection.

It is important to note that the details of input files, run settings and output files all are stored within the *CcpNmr Analysis* project, enabling effective management of multiple structure-calculation runs and the option to re-run the exact same calculation after inspection and refinement of the data.

Structure calculations using *ARIA* are launched in a similar fashion: after selection of the desired restraints the calculation can be launched locally or *via* the CCPNGrid service. *ARIA* calculations are typically more CPU-intensive than *CYANA* calculations and it is often convenient to submit these to a computational cluster. Again, the export and re-import of the required files is performed within a single popup window, making the process simple and robust.

### 2.3. Analysing results   

**Figure 2:**
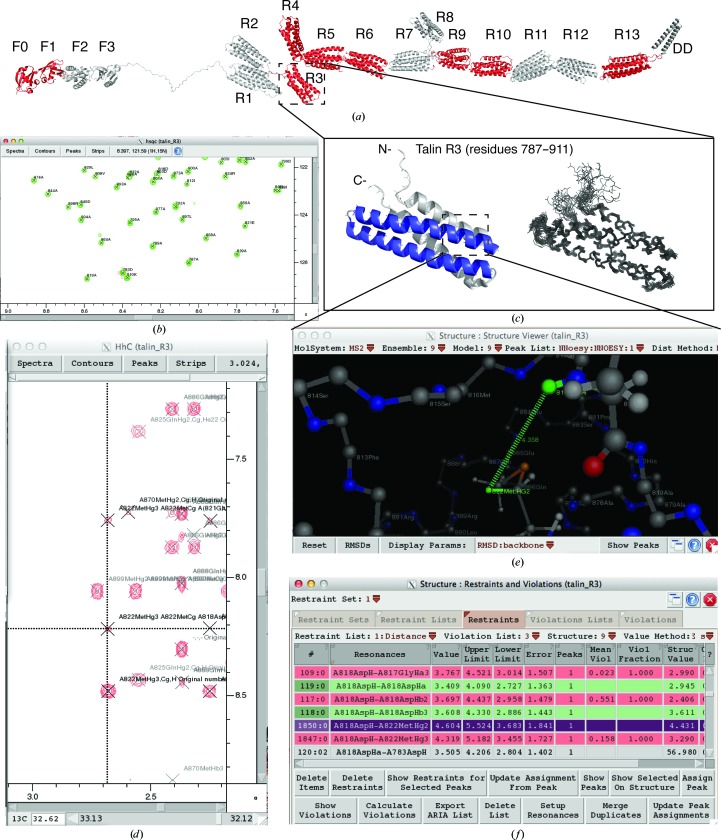
An example of a *CcpNmr Analysis* project for the R3 talin rod domain. (*a*) Structural model of full-length talin showing all 18 talin domains. The structures of the domains shown in red were solved using the *CcpNmr Analysis* pipeline described in this report. (*b*) The fully assigned ^1^H–^15^N HSQC spectrum of R3. (*c*) The structure of the R3 talin rod domain. Left, ribbon drawing of a representative low-energy structure showing the overall topology of the four-helix bundle. The two vinculin binding helices are shown in blue. Right, superimposition of the 20 lowest energy structures consistent with the NMR data. (*d*–*f*) Screenshots of the three windows used to analyse and validate the results of the structure calculation. (*d*) The originating peak in the ^13^C HSQC-NOESY experiment; (*e*) the restraint shown on the structure colour-coded by the size of the violation; (*f*) the table of restraints, colour-coded by the fraction of structures violated. Colours range from green (satisfied) to red (violated).

There are a plethora of tools available within the *CcpNmr Analysis* program for inspection of the structure-calculation results and assessment of the quality and validity of the structure ensemble. The agreement between experimental data and structural results, a so-called violation analysis, is an often-used starting point. The calculation of the violations for any restraint list is achieved with a single click in *CcpNmr Analysis*. In analogy to the *CING* colour-coding scheme (Doreleijers *et al.*, 2012 ▶), all restraints are then colour-coded either green (no violation), yellow, orange or red (severe violation) (Figs. 2 ▶ *e* and 2 ▶ *f*), depending on the size and the prevalence of violations. Note that the *CcpNmr Analysis* criteria for the different colour categories are not identical to those of *CING*. All restraints can be easily visualized on the calculated structure ensemble by using the built-in structure-viewer tool (Fig. 2 ▶ *e*). An essential part of this validation and refinement is the ability to return to the original data, the NOE peaks, and from these violations it is possible to navigate to the specific peak with a single click (Fig. 2 ▶ *d*). This tool also enables the calculation and visualization of either backbone or all-atom r.m.s.d. from the mean of the structural ensemble. Superposition is performed by a parameter-free iterative r.m.s.d.-weighted algorithm based on the singular value decomposition method for optimizing coordinate rotations (Kabsch, 1976 ▶, 1978 ▶), and the final superposition is currently to the structure closest to the mean. In future versions of *CcpNmr Analysis* this will be changed to superposition on the medoid structure, in accordance with the practice mandated by the wwPDB NMR Validation Task Force (Montelione *et al.*, 2013 ▶). With a relevant structure or structure ensemble present in a *CcpNmr Analysis* project, synthetic NOE peak lists can be generated from the structure, for example to determine the completeness of assignment or to compare predicted peaks with the actual experimental data.

*CcpNmr Analysis* also has sophisticated tools for assessing assignment quality. The ‘Test Shift Match’ facility provides information on how many peaks in a spectrum are already assigned to chemical shifts and how many peaks can be converted into restraints, with an annotation for ‘unmatchable’ peaks. This is a very efficient manner of determining missing assignments or identifying peaks that may have been picked erroneously, thereby reducing potential sources of error in the structure calculations. The ‘NOE Contributions’ tool uses the matching of chemical shifts to peak positions to suggest assignments for individual peaks, optionally based upon a structure. The tool identifies and optionally eliminates assignment possibilities where atoms would be too far apart in space to give a contribution to a signal. This can assist in the elimination of erroneous assignments caused by overlapping peaks and potentially reduce the number of possible assignments to a given peak. Moreover, predicted peak locations can be helpful in explaining potential contributions to signal intensities.

### 2.4. Structure validation   

It is good practice to perform validation of a structural ensemble during the multiple cycles (see Fig. 1 ▶) of the structure-determination process (Vuister *et al.*, 2014 ▶) in order to assess whether the calculated ensemble is reasonable in terms of prior physico-chemical knowledge and to find any potential errors in the data set. *CcpNmr Analysis* provides interfaces to two software packages for this task, namely a Python implementation of *RPF* (*PyRPF*; Huang *et al.*, 2012 ▶) and an integrated interface to the iCing validation server (Doreleijers *et al.*, 2012 ▶). *PyRPF* assesses how well a query structural ensemble fits experimental NOESY peak lists and resonance assignment data by calculating so-called RECALL, PRECISION and F-MEASURE (RPF) scores. It also calculates discrimination power (DP) scores, which estimate the difference in F-MEASURE scores between the query structure and ‘random-coil’ structures, as an indicator of the correctness of the overall fold.

*CING* (*Common Interface for NMR structure Generation*) takes assignments, peak lists, restraint lists and structural data as input, which can be submitted as the complete *CcpNmr Analysis* project, and feeds this information to a collection of 21 different validation programs and routines. *CING* then provides an integrated report on the basis of all of these results and an assessment of how valid the structure is on the whole. *CING* calculations are performed on a remote server, with the full results available as highly integrated web pages, and the core results are imported back into *CcpNmr Analysis* once the calculation is complete. Based upon the analysis of the output of the validation routines and external programs, a decision has to be made to either accept the structure or to engage in a further round of refinement (see Fig. 1 ▶). The reasons for a poor-quality structure can be diverse and can originate from any of the previous steps, potentially requiring a re-evaluation of the underlying NMR data, corrections of errors or adjustment of the parameters used to derive the structural restraints.

### 2.5. Deposition of results   

Deposition, both of experimental data (chemical shifts and restraints) in the BMRB repository and structural coordinates in the PDB repository, is generally mandatory before publication of the results of an NMR-based structural study. The ‘Database Deposition’ popup within *CcpNmr Analysis* enables easy preparation of these data. Within this interface, all of the information required for data deposition and present within the *CcpNmr Analysis* project is selected and made available for deposition. Any missing information can be entered within this interface or by using the appropriate popup within *CcpNmr Analysis*. This streamlined facility (Penkett *et al.*, 2010 ▶) assists with the efficient and accurate production of NMRSTAR 3.1 files for BMRB deposition and coordinate files for deposition in the PDB.

### 2.6. Data tracking   

In many NMR structure-generation projects, the number of spectra, peak lists, structures and restraint lists can be very large and a convenient way to visualize all the contents of such a project is often necessary. Within *CcpNmr Analysis*, the project summary contains all this information in one table. Each section of this table can be separately exported in comma-separated or tab-separated formats and the entire table can be exported to a web browser for easy viewing of all data and for export to PDF or PostScript formats.

## 3. Further usage of analysis   

In addition to structural analysis, there are many more ways in which NMR can play a key role in the understanding of the biological system under study, and a *CcpNmr Analysis* project can be organized to contain and analyse all of these data.

(i) *Identification of domain boundaries*. By biochemically varying the boundaries of the protein fragments and recording (^15^N–^1^H) HSQC spectra, *CcpNmr Analysis* allows the easy comparison and documentation of a series of spectra.
(ii) *HSQC analysis* to confirm that engineered protein variants to disrupt interactions or modulate biophysical properties are correctly folded. *CcpNmr Analysis* facilitates the rapid assignment of altered forms *via* transfer of existing assignments to a new molecular system.
(iii) *HSQC titrations to identify interactions between two species*. Chemical shift perturbations provide a simple residue-specific or even atom-specific mapping of binding surfaces onto a known or a homologous structure. *CcpNmr Analysis* allows easy assessment of the perturbation data and has tools to fit binding curves and derive binding constants from chemical shift titration data.
(iv) **HADDOCK* docking to generate testable models of the potential complex*. *CcpNmr Analysis* has a dedicated module for the setting up and execution of *HADDOCK* (Dominguez *et al.*, 2003 ▶) calculations. All required input data are exported in the correct formats, the input scripts are created and the calculation can be executed on a local machine or using the *HADDOCK* server (http://www.nmr.chem.uu.nl/haddock).
(v) *Relaxation studies to probe the dynamics of the system*. Whilst beyond the scope of this report, *CcpNmr Analysis* has integrated modules to extract all of the required parameters (*T*_1_, *T*_2_ *etc.*) from the NMR data contained within a CCPN project.

## 4. Support for the NMR community and software developers   

The Collaborative Computational Project for NMR (CCPN; http://www.ccpn.ac.uk) is a public nonprofit project that serves the macromolecular NMR community through its *CcpNmr* software suite, by collaborative software development and by outreach activities. CCPN also aims to provide a means to integrate existing NMR software within a unified system. The *CcpNmr* software suite encompasses the programs *CcpNmr Analysis* (Vranken *et al.*, 2005 ▶; Stevens *et al.*, 2011 ▶) described here, *CcpNmr ChemBuild* for the generation of NMR-aware molecular topologies of small molecules, *CcpNmr FormatConverter* (Vranken *et al.*, 2005 ▶) for the conversion between 30+ different NMR formats, *CcpNmr NmrScreen* for small-molecule ligand screening by NMR and the *CcpNmr Workflow-Management System* (Wassenaar *et al.*, 2011 ▶) for the management and submission of NMR structure calculations.

The CCPN project also actively promotes the development and spreading of knowledge and best practice in NMR through the organization of meetings, workshops and the development of freely available tutorials. CCPN has participated in a number of collaborative efforts for software integration, such as ExtendNMR (http://www.extend-nmr.eu) and WeNMR (http://www.wenmr.eu), and is actively promoting the use of and integration with CCPN software through workshops for programmers and one-to-one collaborations.

The organization of CCPN is governed by the CCPN Charter. Strategic decisions are taken by the Executive Committee comprised of members of the NMR community, who are are chosen by the CCPN Assembly and typically serve three-year terms. CCPN actively collaborates with other Collaborative Computational Projects in related areas, such as CCP4 for macromolecular crystallography, CCP-EM for electron microscopy, CCPN-NC for NMR–crystallography and CCP-BioSim for biomolecular simulations.

The *CcpNmr* suite of programs is open-source and written principally in Python, and therefore its components can serve as templates for further software development. The program is based entirely on the CCPN data model with its associated subroutine libraries, which support data access, I/O and backwards compatibility and ensure consistency within the data and with the underlying model. Any two programs that both interact independently with the data model seamlessly can share their data. The data model and its application program interface (API; Fogh *et al.*, 2006 ▶) come with copious auto-generated documentation, which is automatically synchronized with the latest version of the model. The same website includes programmers’ tutorials with examples of scripts and the documentation for *CcpNmr Analysis* and its high-level subroutine libraries (beyond the data-model API). All data contained within the *CcpNmr Analysis* program are accessible from the command line, and the user interface refreshes automatically to reflect the data state. This holds true both for scientific data (such as sequences and peaks) and for graphics data, including window positions and contour colours. As a result, the program is completely accessible to other programmers.

## 5. Case study using *CcpNmr Analysis*: talin   

The optimized pipeline described here has significantly improved the efficiency of NMR structure calculation, making it possible to undertake ambitious projects. One such project was talin, a large 2541-amino-acid dimeric protein that plays a key role in regulating cell adhesion and migration.

Talin contains an N-terminal head region (∼50 kDa) that is linked to a ∼220 kDa flexible rod made up of 62 amphipathic helices (Fig. 2 ▶ *a*). The talin rod has a large number of binding partners, including RIAM, vinculin, actin, integrin, synemin, DLC1 and also talin itself, each binding to different regions. Structural knowledge of the domain structure of talin is essential for the understanding of its function. NMR, in conjunction with circular dichroism, small-angle X-ray scattering and X-ray crystallography, enabled us to determine the correct domain boundaries of talin (Fig. 2 ▶ *a*) and purification protocols to produce them (Banno *et al.*, 2012 ▶). This integrated approach allowed us to complete the structures of all 18 domains of talin, nine of which were solved by high-resolution NMR spectroscopy and nine by X-ray crystallography. Together, it has enabled us to build a model of full-length talin (Goult *et al.*, 2013 ▶).

*CcpNmr Analysis* made it possible for a single scientist to determine such a large number of NMR solution structures by streamlining the structure-determination process. Fig. 2 ▶ shows an *CcpNmr Analysis* project for the third talin rod domain, R3, a 124-residue four-helix bundle (Fig. 2 ▶ *c*). R3 is the initial mechanosensing domain in talin, unfolding in response to a low force exerted on talin (Yao *et al.*, 2014 ▶) during initial adhesion assembly. The *CcpNmr Analysis* project for R3 contains all of the spectra required for backbone assignment [the triple-resonance experiments HNCO, HN(CA)CO, HNCA, HN(CO)CA, CBCA(CO)NH and HNCACB], side-chain assignment [H(C)CH-TOCSY and (H)CCH-TOCSY], NOESY spectra [three-dimensional ^15^N-edited NOESY-HSQC (800 MHz, 100 ms), ^13^C-edited NOESY-HSQC (800 MHz, 100 ms) and ^13^C-edited NOESY-HSQC (800 MHz, 80 ms) on aromatics] and HSQC titration series of its interactions with the R3 interaction partner RIAM. This organization of the data into a single project enabled the easy tracking and organizing of all of these spectra.

The backbone assignment was completed using the built-in *CcpNmr Analysis* routine ‘Protein Sequence Assignment’ and the assigned (^1^H,^15^N) HSQC spectrum is shown in Fig. 2 ▶(*b*). After completion of the side-chain assignments, the structure calculation was initiated from within *CcpNmr Analysis* using the built-in *CYANA* interface with unassigned NOESY peak lists picked semi-automatically from the ^15^N-edited NOESY-HSQC (1940 peaks), the ^13^C-edited NOESY-HSQC (3697 peaks) and the ^13^C-edited NOESY-HSQC on aromatics (64 peaks). Dihedral angle restraints generated by the program *DANGLE* from the chemical shift assignments (198 angles) were also used in the calculation. During the course of the *CYANA* calculation, ∼95% of the NOESY peaks were successfully assigned, with 63% of these being short-range, 22% medium-range and 14% long-range assignments. The re-imported structure, assigned peak lists (Fig. 2 ▶ *d*) and generated restraints were then checked for violations (Fig. 2 ▶ *f*) and visualized on the imported ensemble of structures (Fig. 2 ▶ *e*). Fig. 2 ▶(*d*) shows the corresponding NOE peak to a violated restraint for verification of its quality. Following manual inspection of the violations and the output generated by the *CING* program (Doreleijers *et al.*, 2012 ▶), the refined restraints and assignments resulting from this process were taken forward and this process was repeated until the structure was deemed good based on the validation criteria. The addition of the *CING* analysis to this pipeline significantly simplified the refinement process, enabling the easy pinpointing of regions of the calculated structures that were distorted and potentially problematic and thus required closer inspection. Usually, such distortions in the structures arose from errors in the interpretation of the data, such as incorrect or missing resonance assignments, inadvertently picked artefacts or noise peaks in NOESY spectra, or overlapped peaks that distorted the derived distances. More generally, and not exclusive to the R3 project, problems have also been shown to originate from dynamic effects, typically in flexible loop regions, that result in overly restrictive dihedrals or distance restraints. By careful inspection of regions flagged as problematic by the validation software, in conjunction with the experimental data, we were able to recognize and remedy such problems. A total of eight of these refinement cycles of structure calculation, validation and data inspection were required to obtain the final structure of the R3 rod domain. Statistical parameters for the R3 structure are listed in Supplementary Table S1. The R3 structure was deposited in the PDBe repository using the CCPN project file and integrated PDBe interface (Penkett *et al.*, 2010 ▶) as PDB entry 2l7a (Goult *et al.*, 2013 ▶).

Solving protein structures using NMR can often still be a time-consuming process. However, the *CcpNmr Analysis* pipeline described above significantly reduced the time required for the talin project. The total time taken to obtain the R3 structure was approximately three months of calendar time, with one person working part-time on the project. This included the measurement time for six triple-resonance experiments for backbone assignment (6 d on a 600 MHz spectrometer), two TOCSY experiments for side-chain assignment (4 d on a 600 MHz spectrometer) and three NOESY experiments to obtain the distance restraints required to solve the solution structure of the domain (8 d on an 800 MHz spectrometer). Processing of the NMR time-domain data and assignment of the backbone nuclei took ∼3–4 d, the side-chain assignment took ∼10–14 d and the structure calculations, iterative refinement and validation of the structure required approximately one month.

The protocol described here was used for all nine of the NMR solution structures of the talin domains, each taking a similar duration of approximately three months. Structural genomics consortia have quoted durations of approximately one month for determining the structures of proteins of up to 20 kDa as being feasible (Liu *et al.*, 2005 ▶). For the R3 protein, however, we recorded significantly more NMR experiments than proposed by Liu and coworkers, as this improved the reliability of the whole process. Together with the afore­mentioned eight cycles of iterative structure calculation and refinement, we obtained a high-quality, expert-vetted NMR structure. Importantly, as the individual *CcpNmr Analysis* projects of each of the domains save the exact state of the project and contain an exact account of all the data used, the whole structure-determination process was executed in a structured and thoroughly documented way, thus facilitating a proper and unambiguous deposition of the final results in the BMRB and PDB databases, including the required metadata. The NMR data and structures of all of the talin domains were deposited in these two databases, respectively (for details, see Goult *et al.*, 2013 ▶).

## 6. Conclusions and outlook   

*CcpNmr Analysis* v.2 has greatly simplified the practice of NMR-based structure determination. *CcpNmr Analysis* v.2 will be supported in the long term and CCPN v.2 data files will remain fully readable in future versions.

Meanwhile, *CcpNmr Analysis* v.3 is under development. The adoption of modern cross-platform graphics libraries (Qt) facilitates major improvements in the user interface, with support for drag-and-drop and the use of platform-specific styles. The user interface of *CcpNmr Analysis* v.3 will concentrate on supporting common tasks in the simplest possible manner, with separate advanced functionality to give the full, detailed control needed for uncommon and more complex tasks.

In view of the numerous features now incorporated into *CcpNmr Analysis* v.2, v.3 will be divided into components specialized for different tasks, such as spectrum viewing, assignment, structure generation, data extraction and analysis, metabolomics or drug discovery. *CcpNmr Analysis* v.3 is designed to be easily adaptable and extendable not only by programmers but also by spectroscopists: data can now be accessed through a simplified, more user-friendly layer of function calls, and the command flow of the program is echoed to a console, where it can be inspected and used as a template for the generation of user macros. Users will also be able to customize the interface to suit their particular workflows and to easily share these customizations with others. We plan to release the first beta test versions of *CcpNmr Analysis* v.3 in early 2015.

## Supplementary Material

Restraint and structural statistics table.. DOI: 10.1107/S1399004714026662/ba5228sup1.pdf
