# Supplementary material for: Structure calculation, refinement and validation using CcpNmr Analysis
Source: Acta Crystallogr D Biol Crystallogr. 2015 Jan 1;71(Pt 1):154–61. doi: 10.1107/S1399004714026662 (PMC4304695; doi:10.1107/S1399004714026662)
Supplement: Supplementary file 1 [file d-71-00154-sup1.pdf]

BIOLOGICAL CRYSTALLOGRAPHY

Volume 71 (2015)

Supporting information for article:

Structure calculation, refinement and validation using *CcpNmr Analysis*

Simon P. Skinner, Benjamin T. Goult, Rasmus H. Fogh, Wayne Boucher, Tim J. Stevens, Ernest D. Laue and Geerten W. Vuister

Table S1 Solution Structure Determination of Talin R3 (Goult et al., 2013)

	R3 (787-911)
Restraints	
Unique/Ambiguous NOEs	3444/391
Intra-residue	1279/91
Sequential	749/76
Short range (1< [i – j] <5)	841/123
Long range ([i – j] >4)	575/101
φ/ψ dihedral angles[a]	194
Energies (kcal mol^{-1})[b]	
Total	-5480.8 ± 74.8
Van Der Waals	-1088.7 ± 13.9
NOE	31.2 ± 4.8
RMS deviations[b]	
NOEs (Å)	0.013 ± 0.001
(no violations >0.5 Å)	
Dihedral restraints (º) (no violations >5º)	0.31 ± 0.05
Bonds (Å)	0.003 ±0.0001
Angles (º)	0.40 ± 0.01
Impropers (º)	1.22 ± 0.07
Ramachandran map analysis[c]	
Allowed regions	95.5%
Additional allowed regions	4.2%
Generously allowed regions	0.1%
Disallowed regions	0.1%
Pairwise rms differences (Å)[d]	
All Residues	0.45 (0.86)
Secondary Structure	0.40 (0.84)

[a] From chemical shifts using Talos.

[b] Calculated in ARIA 1.2 for the 20 lowest energy structures refined in water.

[c] Obtained using PROCHECK-NMR.

[d] For backbone atoms; value for all heavy atoms in brackets.

Reference

Goult, B. T., Zacharchenko, T., Bate, N., Tsang, R., Hey, F., Gingras, A. R., Elliott, P. R., Roberts, G. C. K., Ballestrem, C., Critchley, D. R. et al. (2013). *J. Biol. Chem.* **288**, 8238–8249.
